# NTCP and Beyond: Opening the Door to Unveil Hepatitis B Virus Entry

**DOI:** 10.3390/ijms15022892

**Published:** 2014-02-19

**Authors:** Koichi Watashi, Stephan Urban, Wenhui Li, Takaji Wakita

**Affiliations:** 1 Department of Virology II, National Institute of Infectious Diseases, 1-23-1 Toyama, Shinjuku-ku, 162-8640 Tokyo, Japan; E-Mail: wakita@nih.go.jp; 2 Department of Infectious Diseases, Molecular Virology, University Hospital Heidelberg, Im Neuenheimer Feld 345, D-69120 Heidelberg, Germany; E-Mail: stephan.urban@med.uni-heidelberg.de; 3 German Center for Infection Research, Heidelberg University, Im Neuenheimer Feld 345, D-69120 Heidelberg, Germany; 4 National Institute of Biological Sciences, No.7 Science Park Road, ZGC Life Science Park, Changping, 102206 Beijing, China; E-Mail: liwenhui@nibs.ac.cn

**Keywords:** HBV, infection, entry, replication, NTCP, SLC10A1, transporter, DMSO, myrcludex-B, cyclosporin

## Abstract

Chronic hepatitis B virus (HBV) infection, affecting approximately 240 million people worldwide, is a major public health problem that elevates the risk of developing liver cirrhosis and hepatocellular carcinoma. Given that current anti-HBV drugs are limited to interferon-based regimens and nucleos(t)ide analogs, the development of new anti-HBV agents is urgently needed. The viral entry process is generally an attractive target implicated in antiviral strategies. Using primary cells from humans and *Tupaia belangeri*, as well as HepaRG cells, important determinants of viral entry have been achieved. Recently, sodium taurocholate cotransporting polypeptide (NTCP) was identified as an HBV entry receptor and enabled the establishment of a susceptible cell line that can efficiently support HBV infection. This finding will allow a deeper understanding of the requirements for efficient HBV infection, including the elucidation of the molecular entry mechanism. In addition, pharmacological studies suggest that NTCP is able to serve as a therapeutic target. This article summarizes our current knowledge on the mechanisms of HBV entry and the role of NTCP in this process.

## 1. Introduction

**Figure 1.**
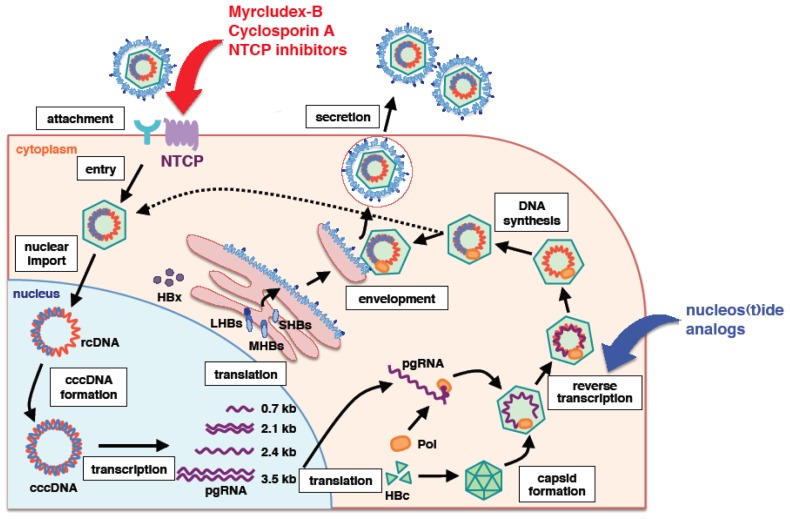
Schematic representation of the hepatitis B virus (HBV) lifecycle. Nucleos(t)ide analogs inhibit reverse transcription. Myrcludex-B, cyclosporin A and some NTCP inhibitors can inhibit the viral entry process by targeting NTCP.

Hepatitis B virus (HBV) infection constitutes a serious public health problem, affecting approximately 240 million carriers worldwide [1]. Chronic HBV infection significantly elevates the risk for developing liver cirrhosis and hepatocellular carcinoma. Currently, conventional interferon-α (IFNα) or PEGylated-IFNα and nucleos(t)ide analogs are available as anti-HBV agents [2,3]. However, IFN-based therapies, which cause significant side effects, yields long-term clinical benefits in less than 40% of treated patients [4]. Nucleos(t)ide analogs suppress an essential step in virus replication and thereby provide biochemical and histological improvement, but some of the early drugs give rise to drug-resistant viruses, which adversely affect long-term clinical outcome. Thus, in order to approach curative treatments, new anti-HBV agents targeting different molecules involved in HBV infection and propagation are needed. Nucleos(t)ide analogs suppress HBV replication mainly by inhibiting the reverse transcription process in the viral lifecycle (Figure 1) [3]. IFN functions as an immunomodulator and is also reported to directly interfere with HBV replication at multiple steps of the lifecycle [5]. Given that HBV encodes only one viral protein carrying enzymatic activity, polymerase, in its genome, strategies for inhibiting viral enzyme are limited. Although capsid or envelope protein assembly and the regulatory X-protein are possible future targets, it is critical for developing new classes of anti-HBV agents to identify cellular factors serving as possible drug targets.

In general, the viral entry step is an attractive target for the development of antiviral agents [6–8]. The early HBV lifecycle, including the entry step, has gained significant attention very recently with regard to molecular mechanisms, triggered by the identification of sodium taurocholate cotransporting polypeptide (NTCP) as a cellular entry receptor [9]. This article summarizes the molecular evidence related to HBV entry, mainly focusing on recent findings, and its implications.

## 2. PreS1 Region of HBV Surface Protein Is Essential for Viral Entry

HBV infection into host hepatocytes follows a multiple step process: (1) initially, HBV reversibly attaches to host cell surface proteoglycans with a low affinity; (2) this is followed by the process involving more specific receptor(s) with high affinity to mediate the early entry step; and (3) after endocytosis-mediated internalization, the virus fuses with the cellular membrane compartment, probably in an endosomal compartment, although the mechanisms are not fully understood. Both initial attachment and, probably more importantly, specific receptor recognition contribute to host specificity and tissue tropism [10]. The initial attachment step is at least partly mediated by heparan sulfate proteoglycans [11–14]. The third internalization step is reported to involve caveolae-, clathrin- or macropinocytosis-dependent endocytosis, depending on the cell types and experimental systems [15–18]. However, cellular factors involved in the high-affinity binding and the early entry process remained to be elucidated until recently.

The HBV surface proteins are composed of three proteins, termed the large (LHBs), middle (MHBs) and small (SHBs) surface proteins, and include the preS1, preS2 and S regions: LHBs encompasses the preS1, preS2 and S regions; MHBs encompasses the preS2 and S; and SHBs comprise the S region [19,20]. The molecular requirement of HBV envelope proteins for HBV infection has been studied for more than a decade using primary hepatocytes from humans and *Tupaia belangeri*, as well as HepaRG cells [21]. A series of analyses using neutralizing antibodies and introduced point mutations suggested that the S and preS1, but not the preS2, regions play a significant role in HBV infection [22–27]. In a direct approach, the preS1 region in the LHBs has been shown to be essentially involved in the HBV infection process. This was demonstrated by the introduction of mutations in the viral context, infection competition with anti-preS1 antibodies and with peptides mimicking this region [28–34]. A myristoylated peptide encompassing amino acids 2–48 of the preS1 region turned out to be the most efficient in infection inhibition of HBV and also the envelope protein-related hepatitis D virus (HDV) [30,31]. Such a peptide has been used as a tool for characterizing the early infection step, including the identification of NTCP as an entry receptor [9] and as a lead substance (Myrcludex-B) presently in the clinical development (see below) [10,35,36].

## 3. Sodium Taurocholate Co-Transporting Polypeptide (NTCP) as a Bona Fide HBV Receptor

One of the recent milestones in the field in HBV molecular biology is the identification of NTCP as a host entry receptor, as reported by Yan and Zhong *et al.* in late 2012 [9]. By affinity purification and mass spectrometry analysis using an HBV preS1-derived lipopeptide as bait, they identified *Tupaia belangeri* NTCP (tsNTCP) as a cellular factor interacting with this lipopeptide. NTCP is a transporter residing in the basolateral membrane of hepatocytes and is involved in the hepatic uptake of mostly conjugated bile salts (see below). The lipopeptide was confirmed to specifically bind human NTCP (hNTCP), as well as tsNTCP, but surprisingly not crab-eating monkey NTCP (mkNTCP), which correlated with the species specificity of HBV infection: HBV is able to efficiently infect humans and *Tupaia*, but not crab-eating monkey [9]. Interestingly, this result also correlated with the *in vitro* binding activity of the peptide to the respective primary hepatocytes [10] and their *in vivo* hepatotropism [37]. The role of NTCP in the viral infection of HBV, its satellite virus, HDV, and a closely related primate hepadnavirus wooly monkey HBV was further examined by knockdown and overexpression analyses [9,38,39]. siRNA-mediated knockdown of NTCP in primary human hepatocytes (PHH), primary *Tupaia* hepatocytes and differentiated HepaRG cells reduced HBV and HDV infection, while ectopic expression of NTCP conferred HBV susceptibility in HepG2 cells, which originally did not support efficient infection [9]. This strongly argues that NTCP is an essential factor for HBV infection. The expression of NTCP in different cells was consistent with the HBV susceptibility, as it was significantly expressed in HBV-susceptible cells, PHH and differentiated HepaRG cells, but was weakly expressed or absent in HepG2, Huh-7, FLC4 and HeLa cells, which show little to no infection [40–42]. The introduction of NTCP into Huh-7 and undifferentiated HepaRG cells conferred HBV infection to these cells to some extent [38]. Although the total expressions in these transduced cells were comparable, hNTCP-expressing HepG2 cells showed much higher infection efficiency when compared with other human hepatocyte cell lines [38,43,44]. In the initial study, infection efficiency was ~10% in NTCP-overexpressing HepG2 cells cultured with medium containing 2% dimethyl sulfoxide (DMSO) [9]. Subsequent analysis showed that increasing the DMSO concentration to more than 2.5%~3% augmented infection efficiency to 50%~70%, as evaluated by immunofluorescence of HBV proteins, although the virus inoculum was different in these studies [38,43]. The speculations include that DMSO augmented the gene expression of NTCP, promoted the membrane localization of NTCP and changed the post-translational modification of NTCP, but the detailed molecular mechanisms for DMSO-mediated promotion of HBV infection is open for further studies. It remains unknown why not all of the cells were infected with HBV in these reports, but it is possible that the NTCP function for supporting HBV entry is reflected by post-translational modification, subcellular localization or other factors that are governed by cell conditions or by more general conditions, such as the cell cycle, cellular microenvironment or architecture. Another open question is on the high susceptibility for HDV, but not HBV, in Huh-7 cells overexpressing hNTCP [9,38]. Future analysis of this issue is necessary in order to establish a cell culture model that is 100% susceptible to HBV infection.

Crucial amino acid sequences in NTCP involved in HBV infection have been analyzed. By sequence comparison between hNTCP and mkNTCP, replacement of amino acids 157–165 of hNTCP with the respective sequence from mkNTCP abrogated the ability to support HBV preS1-binding and, subsequently, infection, while mkNTCP carrying a conversion to this region from hNTCP conferred HBV susceptibility. Thus, amino acids 157–165 of NTCP are crucial for NTCP-mediated HBV binding and infection [9,45]. It has also been shown that hNTCP bearing a substitution of the 84–87 aa from the mouse counterpart was able to bind preS1, but was not functional for HBV infection, while replacing these residues in mouse NTCP (mNTCP) with the human counterparts supported the infection [38,44]. These data indicate that the 84–87 aa residues are a determinant for NTCP function as an HBV entry receptor. It remains to be elucidated why mNTCP does not support HBV infection, but mNTCP was shown to support specific binding of the preS1-lipopeptide on the cell surface, although the binding capacity of mNTCP to the preS1 region appears to be weaker than that of hNTCP [44]. It is possible that the binding of HBV to NTCP is not sufficient and requires an additional molecule or mechanism to trigger the following early infection process.

HDV is a virusoid-like particle, which depends on HBV for assembly and propagation [46]. HDV shares the HBV envelope proteins, LHBs, MHBs and SHBs, and its attachment/early entry mechanism seems to be very similar to that for HBV. Due to its completely different replication strategy, it is very likely that it depends on different cellular factors and follows different pathways after membrane fusion. Intriguingly, HDV infection can be observed by complementing hNTCP in either mouse-derived Hepa1-6, MMHD3 and Hep56.1D cells, rat hepatocyte TC5123 cells or non-hepatocyte HeLa, CHO and Vero cells. This is in stark contrast to HBV, which cannot infect these cells [38,44]. This suggests that HBV requires additional host factors for infection or is restricted at a post-entry step prior to covalently closed circular DNA (cccDNA) formation. It is of particular interest to clarify the molecular mechanisms underlying the different cellular requirements between infection by HBV and HDV, especially when trying to establish a susceptible mouse model in the future.

## 4. Other Factors Essential for HBV Infection?

It is presently unclear whether there are additional cellular factors besides NTCP required for viral infection and determining the tissue and species tropism of HBV. These include factors essentially involved in the viral lifecycle during attachment, internalization, endocytosis, membrane fusion, uncoating, nuclear translocation and cccDNA formation and those affecting post-entry restriction. Overexpression of hNTCP in mouse hepatocyte cell lines, such as Hepa1-6 and MMHD3 cells, did not confer susceptibility to HBV infection, in contrast to the HBV infection observed after NTCP introduction into HepG2 cells [44]. hNTCP conferred efficient HBV infection in HepG2 cells, but only a low efficiency of infection was observed in Huh-7 and undifferentiated HepaRG cells and no detectable infection to mouse and rat hepatoma cells, including Hep56.1D, Hepa1-6 and TC5123 cells [38]. We also showed that different HepG2 clone isolates that similarly expressed high levels of ectopic NTCP, but were likely to have different cellular genetic backgrounds, had diverse efficiencies of HBV infection [43]. These observations favor the existence of additional host factors determining susceptibility to HBV infection. For hepatitis C virus (HCV) infection, multiple cellular factors are required for efficient viral entry, including low density lipoprotein receptor (LDLR), scavenger receptor class B type I (SR-BI), CD81, occludin (OCLN) and claudin-1 (CLDN-1) as viral entry receptors and Niemann-Pick C1-like 1 (NPC1L1), epidermal growth factor receptor (EGFR) and ephrin A2 (EphA2) as other factors involved in entry [21,47,48]. It has been reported that the complementation of both hCD81 and hOCLN are required for rendering high HCV susceptibility in mice [49]. Furthermore, in the case of duck hepatitis B virus (DHBV), multiple factors are suggested to be essential for efficient viral infection. Carboxypeptidase D was confirmed to bind the DHBV envelope and function in viral attachment and entry [50]. However, overexpression of this protein alone in Huh-7 cells did not support DHBV infection [51]. Carboxypeptidase D was able to bind to DHBV and heron HBV, which did not infect primary duck hepatocytes, and this protein is also expressed in non-liver tissues [52]. Thus, additional factors are likely to be required to explain DHBV susceptibility, one candidate of which can include duck NTCP [53]. These examples in viruses that utilize multiple receptors favor pursuing the identification of additional cellular factors crucial for HBV entry.

## 5. General Characteristic Features of NTCP

NTCP, also designated as solute carrier family 10A1 (SLC10A1), is a member of the SLC10 transporter gene family. The SLC10 family consists of seven members (SLC10A1–7). Among these, NTCP and apical sodium-dependent bile salt transporter (ASBT), also known as SLC10A2, are sodium-dependent transporters for bile acids [54]. NTCP is mainly distributed at the basolateral membrane of hepatocytes and plays a major role in the hepatic influx of conjugated bile salts from portal circulation [55,56]. NTCP on the plasma membranes in hepatocytes binds two sodium ions together with one molecule of preferentially conjugated bile salt for uptake. In addition to bile salts, NTCP, like other transporters, binds and/or transports other molecules, including steroid hormones, thyroid hormones, drug-conjugated bile salt and a variety of xenobiotics [57,58]. hNTCP is a 349 aa protein with an apparent mass of 56 kDa and includes a putative seven or nine transmembrane domains with a predicted topology of *N*-terminal extracellular and *C*-terminal intracellular ends [59–61]. While the structure of NTCP has not been resolved, the crystal structures of the ASBTs from *Neisseria* meningitis (ASBT_NM_) and *Yersinia frederiksenii* (ASBT_Yf_) were recently reported [62,63]. ASBT_NM_ shows a ten transmembrane domain and a hydrophobic inward-facing binding cavity. This structure is different from the model for hASBT currently favored based on bioinformatic prediction and experimental data, which carry seven to nine transmembrane helices with *N*-terminal extracellular and the *C*-terminal cytoplasmic domain [60,64,65]. A structural analysis of ASBT_Yf_ proposed two conformations, inward- and outward-open structures of bile salt transporters by rotating two core helices transmembrane (TM)-4 and TM9 [63]. Because ASBT_NM_ and ASBT_Yf_ has only 26% and 22% homology, respectively, with hASBT and even lower homology with hNTCP [62,63], it is uncertain whether the structural features of ASBT_NM_ or ASBT_Yf_ are useful for designing drugs targeting hASBT and hNTCP.

Several single nucleotide polymorphisms (SNPs) that alter the transporter activity of NTCP have been reported [66,67]. As non-synonymous SNPs, I223T, a variant seen in 5.5% of allele frequencies in African Americans, decreased plasma membrane-localized NTCP and reduced its transporter activity. The S267F variant, seen in 7.5% of allele frequencies in Chinese Americans, exhibited almost complete loss of function for bile acid uptake, but possessed normal transport activity for the non-bile acid substrate, estrone sulfate. Another report showed that the A64T and S267F variants, carried by 1.0% and 3.1% of allele frequencies in Koreans, respectively, decreased the uptake of taurocholic acid. These polymorphisms are dependent on ethnicity. However, there have been no reports of serious diseases associated with defects in the NTCP gene. No reports describing NTCP knockout mice have been published to date. Thus, it is difficult to draw conclusions on whether the physiological roles of NTCP are complemented by other factors that share the redundant physiological function and whether NTCP inhibition is able to safely serve as an anti-HBV drug target.

Importantly, it was very recently reported that molecular determinants for the transporter function of NTCP overlapped with those for the ability to support HBV entry [68]. NTCP mutations in amino acids that were critical for bile salt binding (N262A, Q293A/L294A) abrogated both the binding to preS1 peptide and the infection of HBV. The S267F variant of NTCP could neither bind to the preS1 region nor support HBV infection in cell culture.

## 6. NTCP as a Target for Anti-HBV Agents

In general, the viral entry process is an attractive target for the development of antiviral agents. As noted above, the 2–48 aa region of preS1 in the LHBs protein is important for HBV infection [31]. Myrcludex-B, which is an optimized synthetic lipopeptide consisting of the myristoylated 2–48 aa region of preS1, is able to strongly inhibit HBV infection in both cell culture and an *in vivo* mouse model [36]. The IC_50_ in a cell culture model was reported to be approximately 100 pM [35]. Following the successful clinical development of enfuvirtide as the first peptidic HIV entry inhibitor mimicking the region derived from the viral gp41 envelope glycoprotein [69], Myrcludex-B is now under clinical development in phase Ib/IIa [70]. Mechanistically, Myrcludex-B binds hNTCP and inactivates its receptor function for HBV and HDV (Figure 1). Remarkably, IC_50_ to the transporter activity of NTCP was approximately 4 nM [38], showing that binding saturation is not required for receptor inactivation, thus allowing a therapeutic window for infection inhibition without a complete abrogation of bile salt transportation [38]. Thus, agents targeting NTCP are expected to be potent candidates that act as anti-HBV drugs.

Cyclosporin A (CsA) is the first line of such compounds revealed to inhibit HBV infection by targeting NTCP [42,45]. CsA is known to be an immunosuppressant classified as a calcineurin inhibitor and is clinically used for the suppression of the immunological failure of xenografts after tissue transplantation [71]. In cell culture analyses, CsA was also reported to suppress the replication of numerous viruses, including HIV, HCV, influenza virus, severe acute respiratory syndrome coronavirus, human papillomavirus, flaviviruses and HBV [72–79]. In most of these cases, cyclophilins (CyPs), cellular peptidyl prolyl cis-trans isomerases that catalyze conformational changes in proteins and are the primary cellular target for CsA, were critical for efficient viral replication, and CyP inhibition by CsA was responsible for antiviral activity. However, the anti-HBV entry activity of CsA was not mediated by the inhibition of CyP, but rather, via direct targeting of NTCP. CsA bound to NTCP on the plasma membrane and inhibited transporter activity (Figure 1) [42,45]. It also inhibited binding between LHBs and NTCP *in vitro* (Figure 1) [42]. This suggests that CsA interacted with NTCP, thus inhibiting the recruitment of LHBs of incoming HBV to NTCP on the plasma membrane and blocking HBV entry. The anti-HBV activity of CsA was pan-genotypic [42]. Moreover, our derivative analysis identified a series of CsA analogs having a stronger anti-HBV entry activity with a submicromolar IC_50_ [42]. Notably, non-immunosuppressive CsA analogs may be potent anti-HBV agents. Given that non-immunosuppressive CsA analogs, including alisporivir (Debio 025) and SCY-635, have significant activity in decreasing HCV viral load in clinical trials and are regarded as promising anti-HCV drug candidates [80,81], further derivative analysis of CsA may be a reasonable approach for drug development.

As other examples, compounds known to be NTCP inhibitors, including progesterone, propranolol and bosentan, have been shown to block HBV infection (Figure 1) [42]. NTCP substrates, such as taurocholate, tauroursodeoxycholate and bromosulfophthalein, also inhibited HBV infection [38,42,68]. An anticholesteremic drug, ezetimibe, has been shown to block HBV entry [82], and this drug was reported to inhibit the NTCP transporter [83]. These results indicate that compounds modulating NTCP function could substantially inhibit HBV infection. HepG2 cells engineered to overexpress NTCP are also useful for high-throughput screening to identify compounds targeting NTCP and inhibiting HBV infection. One example identified in such chemical screening is the oxysterols, which are oxidized derivatives of cholesterol or by-products of cholesterol biosynthesis [43].

Host-targeting antivirals are generally expected to have significant advantages, including a much lower frequency drug resistance, universal antiviral effects beyond viral genotypes and complementary mechanisms of action that might act in a synergistic manner with currently available antiviral agents [48]. More importantly, they offer an additional therapeutic choice, given that only IFNs and nucleoside analogs are currently available as anti-HBV agents.

## 7. Conclusions

Identification of NTCP as an HBV entry receptor has accelerated the understanding of HBV molecular biology and offered useful experimental systems to analyze the HBV and HDV lifecycle, including the identification of host restriction and dependency factors. NTCP also represents a new therapeutic target in the development of new anti-HBV agents. Further analyses using a new cell culture system are necessary in order to clarify the molecular mechanisms underlying NTCP-mediated HBV infection and to establish an *in vivo* small animal model that fully supports HBV infection.
